# Brain Ct, an Introduction

**Published:** 1986-08

**Authors:** W. D. Jeans


### Book Reviews

**BRAIN CT, AN INTRODUCTION**

**J. R. Bradshaw, Wright, Bristol, 1985.**
**p. 199, ISBN 0–7236–0855–5**
**Price £9.95**

The development by G. Hounsfield of computerised tomographic scanning of the head has not only saved innumerable patients from the miseries of air encephalograms and discomforts of cerebral angiograms, but has made the visualisation of most intra-cerebral lesions, so difficult to see with the older examinations, obvious to the relatively untutored eye. This book is written by one of the two consultant neuro-radiologists at Frenchay Hospital, and uses the experience developed over the last 10 years of using a scanner to teach the reader about CT scans of the head.

It starts by explaining how CT scans are produced and illustrates some of the artefacts commonly seen. The normal anatomy is then demonstrated and the pathology shown. This is presented as a series of cases, each of which has a brief history with the relevant scans and a commentary on the abnormalities shown. The cases are arranged by their CT appearance – high density, low density and so on, with an index by pathology at the end of the book.

The author is well recognised as an excellent teacher by that most critical audience – the radiologists in training. His book demonstrates his skill as a teacher, and like all good teach-yourself texts, moves the reader through the normal into the abnormal by gradual steps so that at the end the majority of abnormalities can be quickly recognised or a likely list of differential diagnoses be considered.

Dr Bradshaw is to be congratulated on producing a book which is so clearly written and which hits its target so accurately. The publishers are to be congratulated on their ability to produce an attractive well-illustrated book at such a reasonable price. Anyone having any reason to look at CT scans or wanting to know about them should purchase a copy and become their own mini-expert.

W. D. Jeans

